# Knowledge and attitude towards home quarantine instructions and associations with history of Covid-19 infection in Malaysia

**DOI:** 10.1186/s12889-024-18739-9

**Published:** 2024-05-14

**Authors:** Shea Jiun Choo, Chee Tao Chang, Huan-Keat Chan, Muhammad Radzi Abu Hassan, Muhammad Hazmi Hamdan, Ai Ting Cheong, Fatin Nadhirah Mat Noh, Nur Syahmina Uzma Mustafa

**Affiliations:** 1 Pharmacy Department, Hospital Taiping, Ministry of Health Malaysia, Taiping, Malaysia; 2 Clinical Research Centre, Hospital Raja Permaisuri Bainun, Ministry of Health Malaysia, Ipoh, Malaysia; 3 School of Pharmacy, Monash University Malaysia, Subang Jaya, Malaysia; 4 Clinical Research Centre, Hospital Sultanah Bahiyah, Ministry of Health Malaysia, Alor Setar, Malaysia

**Keywords:** COVID-19, Home quarantine instructions, Home surveillance order, Knowledge, Practice, Attitude, Warning signs

## Abstract

**Introduction:**

Although COVID-19 has entered the endemic phase, individuals infected with COVID-19 are required to adhere to home quarantine measures. By exploring the public’s knowledge and attitude towards recommended home quarantine measures, their readiness in containing potential COVID-19 outbreak can be determined. This study aimed to assess the public knowledge and attitude towards home quarantine instructions and their association with history of COVID-19 infections.

**Methods:**

This was a web-based cross-sectional study conducted among the public in Malaysia between August to October 2022. All Malaysian adults over 18 years of age were included. Knowledge on home quarantine instructions and COVID-19 warning signs were measured using “True,” “False,” or “I’m not sure”, while attitude towards home quarantine instructions was measured using a five-point Likert Scale. The questionnaire was initially constructed in English and then translated into the national language, Bahasa Malaysia. Face and content validation were performed. The internal consistency of the questionnaire was found to be satisfactory.

**Results:**

1,036 respondents were analyzed, comprised mostly of females (743, 71.6%) with a history of COVID-19 (673, 64.9%). In the knowledge domain, more than 80% of the respondents answered 9 out of 11 home quarantine instructions statements correctly. 457 (44.1%) were unaware or unsure about the minimum distance of the infected individual’s bed from the rest of the occupants in a shared bedroom. The respondents reported relatively weaker knowledge in identifying uncommon warning signs of COVID-19 deterioration, including anuria (162, 44.5%), ingestion problems (191, 52.5%), and immobility (195, 53.6%). In the attitude domain, more than 90% of respondents answered correctly in 8 out of 9 questions. Respondents with a previous history of COVID-19 infections had better knowledge than COVID-19 infection-naïve individuals towards both home quarantine instructions and COVID-19 warning signs.

**Conclusion:**

Most respondents had good knowledge and attitude towards home quarantine instructions, with those previously infected with COVID-19 showing greater awareness of uncommon warning signs. However, there was a notable lack of awareness regarding physical distancing within shared rooms, appropriate disinfectant use and mobility limitation within the household. This study highlights the knowledge gaps to be improved in future educational campaigns.

**Supplementary Information:**

The online version contains supplementary material available at 10.1186/s12889-024-18739-9.

## Introduction

COVID-19 was declared as pandemic by the World Health Organization in March 2020 [1]. The first case of COVID-19 in Malaysia was detected on January 25, 2020, and the number of confirmed cases subsequently hiked [2]. Knowing that transmission of COVID-19 disease is mainly airborne via respiratory droplets and close contact with infected symptomatic cases [3], quarantine of infected individuals has shown to be an effective strategy to break the chain of transmission [4, 5]. In the early stage of the pandemic, individuals who tested COVID-19 positive, regardless of severity, were admitted to the ward or quarantine centres [6].

As Malaysia entered the “transition to endemic” phase since April 2022, the majority of COVID-19 positive individuals no longer needed to undergo involuntary detention. Based on the Ministry of Health Guideline on Home Monitoring and Management of Confirmed COVID-19 case at COVID-19 Assessment Centre, only adult patients clinically diagnosed with COVID-19 category 3 to 5, having uncontrolled comorbidities, immunocompromised, or found to present with warning signs of COVID-19 during evaluation were hospitalized for close monitoring [7]. Whereas, COVID-19 patients that were asymptomatic (Category 1) or minimally symptomatic (Category 2a) were mandated to undergo home surveillance order (HSO) and report their health status twice a day through an electronic home quarantine self-assessment survey on the national contact tracing surveillance application, MySejahtera [7].

As of October 2022, 95.5% of the 23,038 active cases in Malaysia were given HSO [8]. However, there is no direct surveillance and guidance by the health authority on the adherence to home quarantine instructions and monitoring of the warning signs of COVID-19 deterioration during the home quarantine period. In other words, individuals who undergo home quarantine could only rely on their own initiatives to search for information regarding home quarantine instructions and self-disciplines to adhere to them.

A systematic review revealed that overall public knowledge, attitude, and practice regarding COVID-19 were satisfactory across most countries during the early stage of outbreak [9]. Studies conducted in China and Australia indicated that the general public exhibited appropriate practices in adhering to preventive measures [10, 11]. Similarly, local investigations carried out in Malaysia indicated that the public possessed adequate knowledge and displayed positive attitude toward adhering to mitigation strategies aimed at curbing COVID-19 [12, 13]. However, there is a lack of research examining the knowledge, attitude, and practice of the public regarding home quarantine.

As COVID-19 is entering the endemic phase, it is important to educate the public regarding proper home quarantine instructions in accordance to guidelines adopted by the government to prevent future outbreak. In addition, empowering the public in recognizing warning signs of COVID-19 during the period of home quarantine will help them to recognize needs to seek for timely medical attention. This study aimed to assess the public knowledge and attitude towards home quarantine instructions and its associations with history of COVID-19 infections.

## Methods

This was a cross-sectional study conducted among the general public in Malaysia. All Malaysian citizens over 18 years of age were eligible for this study. Those who could not read or understand English or Malay language were excluded.

The study sample size was estimated using the Raosoft sample size calculator (http://www.raosoft.com/samplesize.html). Assuming an infinite study population among the general public of Malaysia, where 50% have good knowledge regarding home quarantine instructions, with 95% confidence level and 5% margin of error, the minimum sample size required was 377.

### Questionnaire

A structured online survey was developed to evaluate the knowledge and attitude towards home quarantine instructions for COVID-19 infections, based on literature and guidelines (6,8–11). The questionnaire primarily focused on: (1) demographic data; (2) knowledge about home quarantine instructions; (3) knowledge about deterioration signs of COVID-19; and (4) attitude towards home quarantine instructions (Supplementary File 1).

The questionnaire was initially constructed in English and then translated into Bahasa Malaysia using a forward-backward-translation approach to ensure linguistic and conceptual equivalence. Face and content validation were performed by a panel of two subject matter experts: one senior academic specialized in public health pharmacy, and one senior clinician specialized in internal medicine. Subsequently, it was pre-tested among five lay persons to examine clarity and relevance. A pilot test was conducted among 30 respondents to check the reliability of the questionnaire. The internal consistency of the questionnaire was found to be satisfactory: Knowledge towards home quarantine instructions (α = 0.618); warning signs of COVID-19 deterioration (α = 0.765); attitude towards home quarantine instructions (α = 0.768) [14].

To measure knowledge about home quarantine instructions and signs of COVID-19 deterioration, the respondents were required to answer whether the item was “True,” “False,” or “I’m not sure.” In terms of attitude, the respondents were asked on a five-point Likert Scale (1 = strongly disagree, 2 = disagree, 3 = neutral, 4 = agree, and 5 = strongly agree) about their likelihood to practice the home quarantine instructions recommended. The overall knowledge and attitude were categorized using Bloom’s cut-off point, as good if the score fell between 80% and 100%, moderate if the score ranged between 60% and 79%, and poor if the score was below 60% [15].

### Data collection

Data collection was performed online using Google Forms. The survey was opened for three months between August and October 2022. The link (URL) of the Google Form online questionnaire was distributed to the potential respondents via social media platforms (e.g., Facebook, Twitter, WhatsApp). To ensure anonymity, subjects were not required to sign in to an account to complete the survey.

The respondents were initially directed to the online Participant Information Sheet (PIS). Further details of the study and consent to participate were given on the PIS webpage. Should they choose not to participate, their intentions were acknowledged, and a “thank you” note expressing the researchers’ respects for the participants’ decision. Should they agree to participate, they were asked to click on a link to proceed with the questionnaire.

### Data analysis

All study variables, including the respondents’ demographic characteristics, knowledge, and attitude, were descriptively analyzed and presented. For the knowledge sections, responses were dichotomized into correct and incorrect/unsure. Responses were categorized and reported as agree and disagree/neutral for the attitude domain. Chi-square tests were used to stratify the knowledge and attitude of the respondents based on their history of COVID-19 infections. The analysis was performed using SPSS version 27; a *p*-value of less than 0.05 was considered statistically significant.

This study was registered with National Medical Research Registry (NMRR), and ethical approval was obtained from Medical Research Ethical Committee (MREC).

## Results

Out of 1,061 responses, nine declined consents, and 15 were excluded due to missing age. Hence, only 1,037 were included in the analysis. The majority of the respondents were between age 30–49 (734,70.8%), female (743, 71.6%), married with at least a child (532, 51.3%), with a bachelor degree (550, 53.0%), with at least one co-morbidities (322, 58.2%), staying with family members (754, 72.7%), with a history of COVID-19 (673,64.9%) and home quarantine (782,75.4%) and completed booster dose (926, 89.3%). The largest source of information regarding COVID-19 was social media (755, 72.8%), followed by health experts (461, 44.5%) and instant messaging applications (450, 43.4%) (Table 1).

**Table 1:** Demographic characteristics of respondents (*n* = 1,037)

	Frequency	Percentage
**Age**	Mean: 36.27 ± 8.74 years	Range: 18–69 years
18–29	231	22.3
30–49	734	70.8
50 and above	72	6.9
**Gender**		
Male	294	28.4
Female	743	71.6
**Ethnicity**		
Malay	745	71.8
Chinese	172	16.6
Indian	47	4.5
Others	73	7.0
**Marital status**		
Married with child	532	51.3
Single	380	36.6
Married without child	90	8.7
Divorce/widow	35	3.4
**Educational level**		
Secondary school	97	9.4
Certificate or diploma	228	22.0
Bachelor degree	550	53.0
Master’s or Ph.D. degree	162	15.6
**Occupation**		
Healthcare related	452	43.6
Non-healthcare related	418	40.3
Students	79	7.6
Housewives	65	6.3
Unemployed/retired	23	2.2
**At least one comorbidities**		
Yes	322	58.2
No	231	41.8
**Household income**		
No income	86	8.3
Less than RM 4,850 per month	385	37.1
RM 4,851 to RM 10,970 per month	375	36.2
More than RM 10,971 per month	191	18.4
**Living status**		
Alone	254	24.5
With family members	754	72.7
With non-family members	29	2.8
**History of COVID-19 positive**		
No	364	35.1
Yes	673	64.9
**History of home quarantine**		
No	255	24.6
Yes	782	75.4
**Vaccination status**		
Never	5	0.5
Completed the first dose	7	0.7
Completed the second dose	99	9.5
Completed at least one booster dose	926	89.3
**Source of information regarding COVID-19**		
Social media	755	72.8
Health experts	461	44.5
Instant messaging applications	450	43.4
Conventional media (print or electronic)	402	38.8
MySejahtera	57	5.5
Ministry of Health websites	13	1.3
Employer or colleagues	11	1.1
Family and friends	6	0.6

The chi-square analysis revealed significant associations between age group (older > younger, *p* < 0.001) and marital status (married > single, *p* = 0.005) with knowledge of home quarantine instructions. However, knowledge showed no significant associations with gender (*p* = 0.140), ethnicity (*p* = 0.385), education level (*p* = 0.482), occupation (*p* = 0.076), household income (*p* = 0.148), living status (*p* = 0.855), history of home quarantine (*p* = 0.819), or vaccination status (*p* = 0.345).

Additionally, gender (female > male, *p* = 0.002) and vaccination status (vaccinated > non-vaccinated, *p* < 0.001) were significantly associated with attitude towards home quarantine instructions. However, attitude showed no significant associations with ethnicity (*p* = 0.814), marital status (*p* = 0.641), education level (*p* = 0.154), occupation (*p* = 0.062), household income (*p* = 0.756), living status (*p* = 0.157), or history of home quarantine (*p* = 0.963).

### Knowledge towards home quarantine instructions

80% of the respondents answered correctly in 9 out of 11 statements related to knowledge towards home quarantine instructions. 220 (21.2%) of the respondents were unaware or unsure whether a shared bathroom should be disinfected with 0.1% Chlorine solution after each use. Moreover, 457 (44.1%) of the respondents were unaware or unsure about the minimum distance of the infected individual’s bed from the rest of the occupants in a shared bedroom (Table 2).

**Table 2:** Knowledge of respondents towards home quarantine instructions (*n* = 1,037)

Statement A person who receives Home Surveillance Order :	Without COVID-19 positive history	With COVID-19 positive history	Total	*p*-value
May not allow visitors.				
Correct Incorrect or unsure	346 (95.1) 18 (4.9)	652 (96.9) 21 (3.1)	998 (96.2) 39 (3.8)	0.140
Needs to maintain physical distance from family members.				
Correct Incorrect or unsure	348 (95.6) 16 (4.4)	655 (97.3) 18 (2.7)	1,003 (96.7) 34 (3.3)	0.325
Needs to limit movement in the house.				
Correct Incorrect or unsure	314 (86.3) 50 (13.7)	594 (88.3) 79 (11.7)	908 (87.6) 129 (12.4)	0.352
Needs to report daily health status through MySejahtera or attend to healthcare providers through phone calls.				
Correct Incorrect or unsure	348 (95.6) 16 (4.4)	662 (98.4) 11 (1.6)	1,010 (97.4) 27 (2.6)	0.008*
May not share eating utensils and personal care products (such as towels).				
Correct Incorrect or unsure	351 (96.4) 13 (3.6)	653 (97.0) 20 (3.0)	1004 (96.8) 33 (3.2)	0.600
Need to comply with basic prevention measures, including wearing a face mask, regular hand washing, and practicing cough etiquette.				
Correct Incorrect or unsure	351 (96.4) 13 (3.6)	653 (97.0) 20 (3.0)	1,004 (96.8) 33 (3.2)	0.600
Should avoid using public transport in case of need to visit the clinic or hospital.				
True False or unsure	331 (90.9) 33 (9.1)	613 (91.1) 60 (8.9)	944 (91.0) 93 (9.0)	0.935
Should have access to a pulse oximeter.				
Correct Incorrect or unsure	319 (87.6) 45 (12.4)	613 (91.1) 60 (8.9)	932 (89.9) 105 (10.1)	0.079
Should stay in a separate bedroom (preferably with an attached bathroom) that is well-ventilated.				
Correct Incorrect or unsure	351 (96.4) 13 (3.6)	662 (98.4) 11 (1.6)	1,013 (97.7) 24 (2.3)	0.048*
If sharing a bathroom, it should be disinfected with 0.1% Chlorine solution after each use.				
Correct Incorrect or unsure	280 (76.9) 84 (23.1)	537 (79.8) 136 (20.2)	817 (78.8) 220 (21.2)	0.281
If sharing a bedroom, the patient’s bed to be put at a minimum distance of three to six feet from the rest of the occupants.				
Correct Incorrect or unsure	214 (58.8) 150 (41.2)	366 (54.4) 307 (45.6)	580 (55.9) 457 (44.1)	0.172

* p<0.05

We performed Chi-square tests and found that respondents who have a history of COVID-19 infection had better knowledge in terms of needs of daily health status reporting (98.4% vs. 95.6%, *p* = 0.008) and room separation requirements (98.4% vs. 96.4%, *p* = 0.048) compared to those who were not infected with COVID-19 before (Table 2).

### Knowledge on warning signs of COVID-19 deterioration

More than 80% of the respondents were able to correctly identify common warning signs of COVID-19 deterioration, including chest pain (894, 86.2%), fever (832, 80.2%), lethargy (876, 84.5%), and shortness of breath (1,003, 96.7%) (Table 3).

**Table 3:** Knowledge on warning signs of COVID-19 deterioration (*n* = 1,037)

Statement The following are warning signs of deterioration of Covid-19 infection:	Without COVID-19 positive history	With COVID-19 positive history	Total	*p*-value
Chest pain				
Correct Incorrect or unsure	308 (84.6) 56 (15.4)	586 (87.1) 87 (12.9)	894 (86.2) 143 (13.8)	0.273
Cough				
Correct Incorrect or unsure	298 (81.9) 66 (18.1)	508 (75.5) 165 (24.5)	806 (77.7) 231 (22.3)	0.018*
Diarrhoea				
Correct Incorrect or unsure	236 (64.8) 128 (35.2)	503 (74.7) 170 (25.3)	739 (71.3) 298 (28.7)	< 0.001*
Fever				
Correct Incorrect or unsure	302 (83.0) 62 (17.0)	530 (78.8) 143 (21.2)	832 (80.2) 205 (19.8)	0.104
Lethargy				
Correct Incorrect or unsure	303 (83.2) 61 (16.8)	573 (85.1) 100 (14.9)	876 (84.5) 161 (15.5)	0.420
Loss of smell sensory				
Correct Incorrect or unsure	302 (83.0) 62 (17.0)	508 (75.5) 165 (24.5)	810 (78.1) 227 (21.9)	0.005*
Loss of taste sensory				
Correct Incorrect or unsure	305 (83.8) 59 (16.2)	508 (75.5) 165 (24.5)	813 (78.4) 224 (21.6)	0.002*
Nausea or vomiting				
Correct Incorrect or unsure	259 (71.2) 105 (28.8)	512 (76.1) 161 (23.9)	771 (74.3) 266 (25.7)	0.083*
Muscle pain				
Correct Incorrect or unsure	274 (75.3) 90 (24.7)	469 (69.7) 204 (30.3)	743 (71.6) 294 (28.4)	0.057
Persistent or worsening conditions such as cough, nausea/vomiting/diarrhoea				
Correct Incorrect or unsure	331 (90.9) 33 (9.1)	634 (94.2) 39 (5.8)	965 (93.1) 72 (6.9)	0.048*
Reduced level of consciousness				
Correct Incorrect or unsure	250 (68.7) 114 (31.3)	509 (75.6) 164 (24.4)	759 (73.2) 278 (26.8)	0.016*
Reduced urine output in 24 h				
Correct Incorrect or unsure	162 (44.5) 202 (55.5)	378 (56.2) 295 (43.8)	540 (52.1) 497 (47.9)	< 0.001*
Shortness of breath				
Correct Incorrect or unsure	351 (96.4) 13 (3.6)	652 (96.9) 21 (3.1)	1,003 (96.7) 34 (3.3)	0.697
Sore throat or flu				
Correct Incorrect or unsure	287 (78.8) 77 (21.2)	493 (73.3) 180 (26.7)	780 (75.2) 257 (24.8)	0.047
Unable to get out of bed without assistance				
Correct Incorrect or unsure	195 (53.6) 169 (46.4)	446 (66.3) 227 (33.7)	641 (61.8) 396 (38.2)	< 0.001*
Unable to take food or drinks				
Correct Incorrect or unsure	191 (52.5) 173 (47.5)	442 (65.7) 231 (34.3)	633 (61.0) 404 (39.0)	< 0.001*

* p<0.05

Respondents with no previous history of COVID-19 infections were more likely to correctly identify loss of smell (83.0% vs. 75.5%, *p* = 0.005) and taste (83.8% vs. 75.5%, *p* = 0.002) as deterioration symptoms compared to those with a history of COVID-19 infections. In contrast, respondents with previous history of COVID-19 infections were more likely to correctly identify atypical symptoms such as diarrhoea (74.7% vs. 64.8%, *p* < 0.001), reduced urine output (56.2% vs. 44.5%, *p* < 0.001), immobility (66.3% vs. 53.6%, *p* < 0.001) and ingestion problems (65.7% vs. 52.5%, *p* < 0.001) as signs of COVID-19 deterioration compared to those without (Table 3).

### Attitude towards home quarantine instructions

More than 90% of the respondents uttered their agreement towards eight out of the nine attitude statements regarding home quarantine instructions. Notably, 97.9% of the respondents agreed to practice all the basic preventative measures during home quarantine, including wearing a face mask, regular hand washing, and practicing cough etiquette. Compared to the other home quarantine instructions, relatively fewer respondents reported agreement (867, 83.6%) in limiting their movement in the house during home surveillance orders (Table 4). There was no significant association in attitude between those with and without COVID-19 history.

**Table 4:** Attitude towards home quarantine instructions

Statement	Without COVID-19 positive history	With COVID-19 positive history	Total	*p*-value
I should stay at home throughout the home quarantine period.				
Disagree or neutral Agree	16 (4.4) 348 (95.6)	37 (5.5) 636 (94.5)	53 (5.1) 984 (94.9)	0.442
I should allow visitors.				
Disagree Agree or neutral	351 (96.4) 13 (3.6)	641 (95.2) 32 (4.8)	992 (95.7) 45 (4.3)	0.372
I should report daily health status through the MySejahtera app.				
Disagree or neutral Agree	21 (5.8) 343 (94.2)	35 (5.2) 638 (94.8)	56 (5.4) 981 (94.6)	0.699
I should limit movement in the house.				
Disagree or neutral Agree	64 (17.6) 300 (82.4)	106 (15.8) 567 (84.2)	170 (16.4) 867 (83.6)	0.447
I should maintain physical distance from other household members.				
Disagree or neutral Agree	10 (2.7) 354 (97.3)	29 (4.3) 644 (95.7)	39 (3.8) 998 (96.2)	0.207
I should practice all the essential preventative measures, including wearing a face mask, regular hand washing, practicing cough etiquette.				
Disagree or neutral Agree	5 (1.4) 359 (98.6)	17 (2.5) 656 (97.5)	22 (2.1) 1,015 (97.9)	0.219
I should use separate eating utensils and personal care products (such as towels) from household members.				
Disagree or neutral Agree	15 (4.1) 349 (95.9)	32 (4.8) 641 (95.2)	47 (4.5) 990 (95.5)	0.639
I should obtain a portable fingertip pulse oximeter and self-monitor oxygen saturation level.				
Disagree or neutral Agree	30 (8.2) 334 (91.8)	60 (8.9) 613 (91.1)	90 (8.7) 947 (91.3)	0.713
I should stay in a separate room as a household member.				
Disagree or neutral Agree	8 (2.2) 356 (97.8)	25 (3.7) 648 (96.3)	33 (3.2) 1,004 (96.8)	0.184

## Discussion

This study represents one of the earliest attempts to evaluate the general public’s knowledge and attitude regarding home quarantine measures in response to COVID-19 endemic. Overall, the majority of respondents exhibited good understanding and positive attitude towards home quarantine instructions. Furthermore, individuals with a prior history of COVID-19 infection notably displayed enhanced knowledge, particularly in areas such as health status reporting and implementing room separation measures throughout the isolation period.

Respondents who were older and married have better knowledge towards home quarantine instructions, although a local study conducted six months after COVID-19 was declared pandemic among a similar population reported better knowledge among middle-aged people [13]. Older individuals and those who were married may have had more exposure to health information and guidelines through traditional media channels or healthcare providers. Additionally, their life experiences and responsibilities, such as caring for families or managing households, may have heightened their awareness and adherence to home quarantine protocols.

Additionally, female and those who were vaccinated have better attitude towards home quarantine instructions. Interestingly, men were less knowledgeable about the symptoms of COVID-19 than women in Pakistan [16]. This gender disparity in knowledge and attitude could be attributed to societal norms which influenced men to downplay health concerns or exhibit a sense of invulnerability, leading to lower levels of knowledge and negative attitude to health protocols. The higher likelihood of positive attitude towards quarantine instructions among vaccinated individuals suggests that this group of respondents took a proactive approach to manage their health and inherently had a greater understanding of the importance of preventive measures in mitigating the spread of COVID-19.

More than two-fifth of the respondents in this study were not aware about the minimum distance between beds in a shared room, revealing knowledge gaps to focus on in future public health education on COVID-19 preventive measures. In fact, a study reported that at least three to six feet away is critical in reducing transmission of COVID-19 [17]. While it is highly advisable for individuals infected with COVID-19 to be isolated, this could be impossible for settings with limited domestic space. In fact, a few clusters involving immigration detention centres, prison, factory dormitories and construction sites were identified for contracting COVID-19 during the pandemic in Malaysia. Hence, health authorities may contemplate the provision of temporary quarantine facilities as a proactive measure to mitigate the risk of outbreaks.

On the other hand, a local study revealed that more than 50% of COVID-19 cases were due to cross-infection among household members [18]. In our study, about one in five respondents were unaware of the need to limit movement in the house. In terms of attitude, we also found a relatively lower level of agreement with this instruction compared to the others. This phenomenon may stem from the belief that individuals under HSO were solely required to refrain from leaving their homes, without any restrictions on mobility within the household. This misconception warrants attention and clear correction, as it has the potential to contribute to cross-infection among household members.

In consistent with previous local and international studies [13, 19, 20], most respondents reported good knowledge of common warning signs of COVID-19 deterioration. Notably, less common warning signs of COVID-19 infection such as diarrhoea, reduced urine output, immobility, and ingestion individuals were better known in respondents with a history of COVID-19. At present, individuals undergoing HSO no longer have direct supervision from healthcare workers and are monitored only through the national contact tracing surveillance application (MySejahtera). This approach relies heavily on community solidarity and responsibility to self-report their infection status and daily wellness. Hence, by filling the information gap concerning COVID-19 worsening indicators, it enables the public to seek medical care in a timely manner.

This study was adequately powered to reflect knowledge and attitude regarding home quarantine instructions in the Malaysian population. Other than determining the preparedness of Malaysians in the era of COVID-19 endemic, it also addressed the knowledge gap in educating the public about home quarantine measures to prevent future outbreak. However, as we did not perform stratified sampling, we observed an underrepresentation of the elderly, men, and non-healthcare workers. Due to data collection time lapse, respondents may inaccurately report their past experiences due to recall bias. Respondent bias may also present as participants might have provided socially desirable responses.

## Conclusion

The respondents demonstrated a good understanding of most home quarantine instructions, with those who had previously contracted COVID-19 showing better awareness of uncommon warning signs COVID-19 deterioration. Generally, participants displayed positive attitude towards home quarantine instructions. However, there was a notable lack of awareness regarding the importance of physical distancing within shared rooms and the necessity of using suitable disinfectants as well as mobility limitation within the household. These underscore the need for future educational campaigns to address the knowledge gaps identified.

### Electronic supplementary material

Below is the link to the electronic supplementary material.

Supplementary Material 1

## Data Availability

The datasets generated and/or analyzed during the current study are not publicly available due to confidentiality of respondents, but are available from the corresponding author on reasonable request.
