# Supplementary material for: Knowledge and attitude towards home quarantine instructions and associations with history of Covid-19 infection in Malaysia
Source: BMC Public Health. 2024 May 14;24:1311. doi: 10.1186/s12889-024-18739-9 (PMC11094920; doi:10.1186/s12889-024-18739-9)
Supplement: Supplementary file 1 — Supplementary Material 1 [file 12889_2024_18739_MOESM1_ESM.docx]

**Supplementary File 1**

**Knowledge And Attitude Towards Home Quarantine Instructions and Associations with History of Covid-19 Infection in Malaysia**

***Please put a tick (✔) next to your answer of choice.***

**Section A: Participant’s demographic**

| **No.** | **Demographic data** |  | **(✔)** |  | **(✔)** |
| --- | --- | --- | --- | --- | --- |
| 1. | State | Federal Territory of Kuala Lumpur, Federal Territory of Labuan, Federal Territory of Putrajaya |  | Perlis |  |
|  |  | Johor |  | Penang |  |
|  |  | Kedah |  | Pahang |  |
|  |  | Kelantan |  | Selangor |  |
|  |  | Malacca |  | Sabah |  |
|  |  | Negeri Sembilan |  | Sarawak |  |
|  |  | Perak |  | Terengganu |  |
| 2. | Gender | Male |  | Female |  |
| 3. | Age (years) |  | | | |
| 4. | Ethnic group | Malay |  | Indian |  |
|  |  | Chinese |  | Others, please state |  |
| 5. | Marital status | Never married |  | Married without child |  |
|  |  | Married with child |  | Divorced/Widowed |  |
| 6. | Highest educational level | No formal education |  | Certificate or diploma |  |
|  |  | Primary |  | Bachelor’s degree |  |
|  |  | Secondary |  | Postgraduate degree: Master or PhD |  |
| 7. | Employment sector | Finance |  | Healthcare |  |
|  |  | Defense |  | Tourism, Arts and Culture |  |
|  |  | Housewife/house husband |  | Housing and local government |  |
|  |  | International trade |  | Sportsman/Sportswoman |  |
|  |  | Education |  | Human Resources |  |
|  |  | Agriculture |  | Entrepreneur |  |
|  |  | Transportation |  | Science, technology and innovation |  |
|  |  | Food and Beverage Industries |  | Communication and multimedia |  |
|  |  | Commodities Industries |  | Student |  |
|  |  | Others, please state |  |  |  |
| 8. | Household income per month | No income |  | RM4,851-RM10,970 |  |
|  |  | <RM4,850 |  | >RM10,971 |  |
| 9. | Living status | Alone |  | With non-family |  |
|  |  | With family |  |  |  |

**Section B: Background relevant to Covid-19 infection.**

| **No** | **Statement** | **Options** | **(✔)** |
| --- | --- | --- | --- |
| 1. | Have you been infected with COVID-19 (positive PCR or RTK test) before? | Yes |  |
|  |  | No |  |
| 2. | Have you ever received a Home Surveillance Order? | Yes |  |
|  |  | No |  |
| 3. | Please indicate your vaccination status. | Not vaccinated |  |
|  |  | Completed first dose |  |
|  |  | Completed second dose |  |
|  |  | Completed third (booster dose) |  |
| 4. | Which of the following is the source of your information regarding home quarantine instructions? | Social media (Facebook, Twitter, Instagram) |  |
|  |  | Instant messaging (WhatsApp, Telegram, WeChat) |  |
|  |  | News (printed/electronic)  *Berita* |  |
|  |  | Healthcare professionals |  |
|  |  | Others |  |

**Section C: Knowledge about home quarantine instructions**

Based on your current knowledge, please indicate if the following statement is true or false regarding home quarantine instructions implemented by the Ministry of Health, Malaysia.

| **No.** | **Statement** | **True** | **False** | **I’m not sure** |
| --- | --- | --- | --- | --- |
| 1. | A person who receives Home Surveillance Order : | | | |
|  | 1. May allow visitors. |  |  |  |
|  | 1. Needs to maintain physical distance with family members. |  |  |  |
|  | 1. Needs to limit movement in the house. |  |  |  |
|  | 1. Needs to report daily health status through MySejahtera or attend to healthcare providers through phone calls. |  |  |  |
|  | 1. May share eating utensils and personal care products (such as towels). |  |  |  |
|  | 1. Does not need to comply with basic prevention measures, including wearing a face mask , regular hand washing and practice cough etiquette. |  |  |  |
|  | 1. Should avoid using public transport in case of need to visit the clinic or hospital. |  |  |  |
|  | 1. Should have access to a pulse oximeter. |  |  |  |
|  | 1. Should stay in a separate bedroom (preferably with an attached bathroom) that is well-ventilated. |  |  |  |
|  | 1. If sharing a bathroom, it should be disinfected with 0.1% Chlorine solution after each use. |  |  |  |
|  | 1. If sharing a bedroom, the patient’s bed to be put at a minimum distance of three to six feet from the rest of the occupants. |  |  |  |
| 2. | Which of the following are **warning signs of deterioration of Covid-19 infection:** (*May tick more than one*) |  |  |  |
|  | 1. Chest pain |  |  |  |
|  | 1. Cough |  |  |  |
|  | 1. Diarrhea |  |  |  |
|  | 1. Fever |  |  |  |
|  | 1. Lethargy |  |  |  |
|  | 1. Loss of smell sensory |  |  |  |
|  | 1. Loss of taste sensory |  |  |  |
|  | 1. Nausea or vomiting |  |  |  |
|  | 1. Muscle pain |  |  |  |
|  | 1. Persistent or worsening of condition such as cough, nausea/vomiting/diarrhea |  |  |  |
|  | 1. Reduced level of consciousness |  |  |  |
|  | 1. Reduced urine output in 24 hours |  |  |  |
|  | 1. Shortness of breath |  |  |  |
|  | 1. Sore throat or flu |  |  |  |
|  | 1. Unable to get out of bed without assistance |  |  |  |
|  | 1. Unable to take food or drink |  |  |  |

**Section D: Attitude towards home quarantine instructions**

Using the rating scale from 1 (strongly disagree) to 5 (strongly agree) please indicate how important or accurate each of the following home quarantine instructions as stated below.

| **No.** | **Statement** | ***1***  ***Strongly disagree*** | ***2***  ***Disagree*** | ***3***  ***Neither agree or disagree*** | ***4***  ***Agree*** | ***5***  ***Strongly agree*** |
| --- | --- | --- | --- | --- | --- | --- |
| 1. | I should stay at home throughout the home quarantine period. |  |  |  |  |  |
| 2. | I should allow visitors. |  |  |  |  |  |
| 3. | I should report daily health status through the MySejahtera app. |  |  |  |  |  |
| 4. | I should limit movement in the house. |  |  |  |  |  |
| 5. | I should maintain physical distance with other household members. |  |  |  |  |  |
| 6. | I should practice all the basic preventative measures including: wear a face mask, regular hand washing, practice cough etiquette. |  |  |  |  |  |
| 7. | I should use separate eating utensils and personal care products (such as towels) from household members. |  |  |  |  |  |
| 8. | I should obtain a portable fingertip pulse oximeter and self-monitor oxygen saturation level. |  |  |  |  |  |
| 9. | I should stay in a separate room as a household member. |  |  |  |  |  |
